# Characterization of the size and location of dyssynchronous regions in patients undergoing CRT

**DOI:** 10.1186/1532-429X-14-S1-P215

**Published:** 2012-02-01

**Authors:** Jonathan Suever, Brandon K Fornwalt, Michael Lloyd, John N Oshinski

**Affiliations:** 1 Wallace H. Coulter Department of Biomedical Engineering, Georgia Institute of Technology / Emory University, Atlanta, GA, USA; 2 Dept of Pediatrics and Biomedical Engineering, Division of Pediatric Cardiology, University of Kentucky, Lexington, KY, USA; 3 Dept of Cardiology, Division of Electrophysiology, Emory University, Atlanta, GA, USA; 4 Dept of Radiology & Imaging Sciences, Emory University, Atlanta, GA, USA

## Background

The amount and location of left ventricular (LV) mechanical dyssynchrony affects an individual’s ability to respond positively to cardiac resynchronization therapy (CRT) [Bax et al JACC 2005]. By using high temporal resolution short-axis cines, it is possible to derive radial motion curves throughout the LV. These radial motion curves can be used to create maps showing dyssynchronous regions in patients enrolled for CRT.

The objective of this study was to characterize the size and location of areas of mechanical dyssynchrony in patients scheduled for CRT by comparing their radial wall motion curves to radial motion curves from normal subjects.

## Methods

CMR was performed in 5 normal subjects with no presence of dyssynchrony (QRS<120ms) and 13 patients scheduled for CRT that met current inclusion criteria (QRS>120ms, NYHA HF class III-IV).

Endocardial borders were traced in normal subjects and patients on high-temporal resolution SSFP short-axis cines (60 frames per cardiac cycle). Radial shortening at 100 locations around the contour relative to the center of mass of the LV was determined for each slice. A *reference* curve was generated for each patient by averaging all curves with a similar trajectory using quality threshold (QT) clustering. The temporal delay between the radial motion curves at each location relative to the *reference* was determined using cross-correlation analysis. By repeating this analysis at all locations throughout the LV, a mechanical dyssynchrony map was generated and projected onto an AHA 17-segment bullseye.

The mean and standard deviation of delay times throughout the LV were determined in the normal subjects. A “normal file” was defined using a 95% confidence interval based on these subjects. By comparing patients to this normal file, we could identify 1) the most dyssynchronous region and 2) the size of this dyssynchronous region.

## Results

In the normal subjects, the normal range of delay times was -49 to 47 ms.

**Figure 1:**
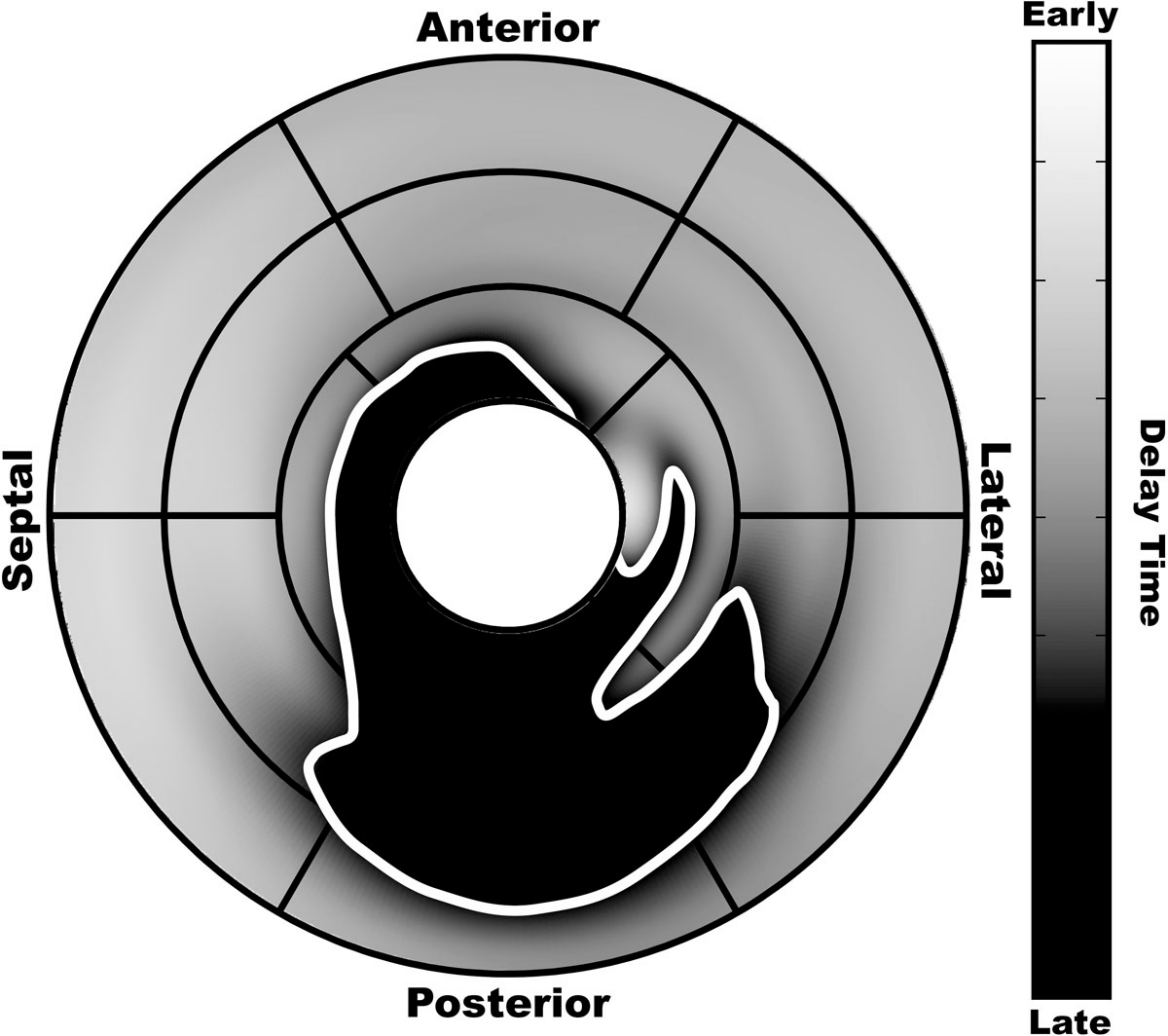
Mechanical dyssynchrony map projected onto the AHA 17-segment model with the dyssynchronous region outlined in white.

The most dyssynchronous region was posterolateral in 6 patients, anterolateral in 4 patients, and septal in 4 patients. The CRT patients had an average of 8.6±1.9dyssynchronous segments which comprised 48.9±12.1 % of the LV, figure 1.

## Conclusions

Using cross-correlation analysis of radial displacement curves from high temporal resolution cine CMR images, regional mechanical dyssynchrony maps can be generated. By comparing patients to a “normal file”, the size and location of dyssynchronous regions in the LV can be determined. These maps could be used for CRT LV lead placement planning.

## Funding

This research was funded by the National Science Foundation Graduate Research Fellowship and the American Heart Association.

